# Diagnostic accuracy of clinical tests for cam or pincer morphology in individuals with suspected FAI syndrome: a systematic review

**DOI:** 10.1136/bmjsem-2020-000772

**Published:** 2020-04-27

**Authors:** Rahel Caliesch, Martin Sattelmayer, Stephan Reichenbach, Marcel Zwahlen, Roger Hilfiker

**Affiliations:** 1 Physiotherapy, HES-SO Valais-Wallis, Leukerbad, Valais-Wallis, Switzerland; 2 Department of Health Professions, Bern University of Applied Sciences, Bern, Switzerland; 3 Institute of Social and Preventive Medicine, University of Bern, Bern, Switzerland; 4 Department of Rheumatology, Immunology, and Allergology, Bern University Hospital, Bern, Switzerland; 5 Graduate School for Health Sciences, University of Bern, Bern, Switzerland

**Keywords:** hip, review, sports physiotherapy, validity

## Abstract

**Objectives:**

To determine the diagnostic accuracy of clinical tests for cam or pincer morphology in individuals with suspected femoroacetabular impingement (FAI) syndrome and to evaluate their clinical utility.

**Design:**

A systematic review of studies investigating the diagnostic accuracy of clinical tests for cam and pincer morphology.

**Data sources:**

PubMed, Embase, CINAHL and SPORTDiscus.

**Eligibility criteria for selecting studies:**

Studies investigating the diagnostic accuracy of clinical tests for cam, pincer or mixed morphology in symptomatic patients. Patients had to undergo an index test and a reference test able to identify cam or pincer morphology. Study results have to allow the calculation of true or false positives and/or negatives to calculate sensitivity, specificity, likelihood ratios (LR) and post-test probabilities.

**Results:**

Eight studies were included, investigating 17 tests and two test combinations. The studies reported a low specificity for all tests, ranging from 0.11 to 0.56. Sensitivity ranged from 0.11 to 1.00, with high sensitivities for the flexion-adduction-internal rotation (FADIR), foot progression angle walking (FPAW) and maximal squat tests. We estimated that negative test results on all of these three tests would result in a negative LR of 0.15. However, we judged the studies to provide low-quality evidence.

**Conclusion:**

There is low-quality evidence that negative test results reduce the post-test probability of cam or mixed morphologies and that consecutive testing with the FADIR, FPAW and maximal squat tests might be used as a clinical test combination. We would not recommend their use to confirm the diagnosis of FAI syndrome.

**PROSPERO registration number:**

CRD42018079116.

**What is already known**

- There is only limited evidence on diagnostic test accuracy for clinical tests to diagnose cam, pincer or mixed morphologies. The most current systematic reviews on clinical tests searched studies up to August 2014 and one review on flexion-adduction-internal rotation searched up to January 2017.

**What are the new findings**

- There is still only low-quality evidence for the diagnostic test accuracy of clinical tests for the detection of cam, pincer or mixed morphologies. A combination of three tests might be the best strategy to exclude a cam or mixed morphology, but this should be confirmed in a future study by a multivariable logistic regression model.

## Introduction

The diagnosis of patients with hip and groin pain is challenging, as there may be multiple underlying aetiologies.^1^ Femoroacetabular impingement (FAI) is a common cause of symptoms, with a prevalence ranging from 18% to 94%.^2^ FAI is characterised by an abnormal morphology of the proximal femur or the acetabulum. This results in premature contact between the femoral head or neck and the acetabulum during hip flexion and rotation, which in turn may lead to labral tears and degeneration of the acetabular cartilage.^3 4^

Three types of anatomical morphologies are known to result in FAI: the cam and pincer morphologies or a mixed form of both.^3^ Early recognition of and intervention for FAI syndrome is needed to reduce sequelae such as osteoarthritis of the hip.^3^ Physiotherapy and activity modification may improve patient-reported outcomes and hip-related quality of life.^5–7^ Therefore, it is important to use adequate diagnostic tests. Arthroscopy, magnetic resonance arthrography (MRA), MRI, CT and radiography are currently used to diagnose cam or pincer morphology. However, these techniques are either invasive, time consuming or expensive.

To avoid unnecessary costs and invasive techniques, several clinical tests have been proposed for the diagnosis of FAI morphology. There are already systematic reviews of the diagnostic accuracy of such clinical tests^1 8–11^; however, they did not distinguish between symptomatic and asymptomatic participants, or between cam/pincer morphology and labral tears. According to a consensus meeting in 2016, the term ‘FAI syndrome’ requires a triad of *symptoms*, *clinical signs* and *imaging findings*; in other words, patients have to complain about pain, show a positive clinical test and demonstrate positive imaging findings of some kind of cam or pincer morphology. Additionally, they may or may not exhibit labral or articular cartilage damage.^12^ Patients included in several previously published studies were asymptomatic or presented only with labral tears without FAI morphology, which is not in accordance with the official definition of an FAI syndrome. Symptomatic labral tears without accompanying cam or pincer morphology are not considered to represent FAI syndrome. Tests conducted in symptomatic patients (‘diagnostic setting’) or in asymptomatic people (‘screening setting’) are performed in two different contexts. Therefore, diagnostic accuracy should be assessed separately in symptomatic and asymptomatic individuals.

The overall aim of this systematic review was to examine the diagnostic accuracy of clinical tests for cam, pincer or mixed morphology in symptomatic patients. Specific aims were to evaluate (1) the sensitivity, specificity and likelihood ratios (LR) of clinical tests for cam or pincer morphology, (2) the clinical utility of these tests, and (3) how clinical tests can be combined to increase clinical utility.

## Methods

The review protocol was registered in the international Prospective Register of Systematic Reviews.

The Cochrane Handbook for Diagnostic Test Accuracy Reviews was used for the conduct of this study and the Preferred Reporting Items for a Systematic Review and Meta-analysis of Diagnostic Test Accuracy Studies (PRISMA-DTA) checklist was used for reporting.^13 14^

The search was conducted from inception to February 2019 in the following electronic databases: PubMed, Embase, CINAHL and SPORTDiscus (see online supplementary file 6 for the PubMed search strategy). Reference lists of included studies were checked for additional articles, and searches for papers cited in the included articles were performed using Google Scholar.

10.1136/bmjsem-2020-000772.supp6 Supplementary data

### Selection criteria

#### Study design

Studies were included if: (1) they investigated the diagnostic accuracy of clinical tests for cam, pincer or mixed morphology of the hip; (2) they included patients with symptoms such as groin, hip or buttock pain; (3) all patients underwent both an index test and a reference test that was able to identify cam or pincer morphology; and (4) the results allowed for the calculation of true or false positives and/or negatives. Studies including asymptomatic individuals and those classifying participants with labral tears only as true positives were excluded. No restrictions were set regarding the language of publication, study settings or the ages or previous surgical histories of included patients.

#### Index tests

Included studies had to describe a clinical test that was intended to identify cam, pincer or mixed morphology of the hip joint. All available clinical tests for FAI were accepted as index tests (ie, flexion-abduction-external rotation (FABER), flexion-adduction-internal rotation (FADIR), maximal squat test, and others).

#### Target condition

Patients had to show imaging findings of cam or pincer morphology,^12^ and could also exhibit labral or articular cartilage damage. Patients presenting with acetabular labral tears without any cam or pincer morphology were not classified as true positives.

#### Reference tests

MRA, MRI, CT and radiography were considered to be adequate reference tests.^12^ See online supplementary table 3S for a short description of the diagnostic accuracies of all reference tests used in the included studies and online supplementary table 4S for the reliability of plain radiography for measuring alpha angles.

10.1136/bmjsem-2020-000772.supp2 Supplementary data

10.1136/bmjsem-2020-000772.supp3 Supplementary data

### Screening process

Titles and abstracts were independently screened by two reviewers (RC and RH), and consensus was sought. The same procedure was applied for full texts of included references; in case of disagreement, a third reviewer decided.

### Data extraction and quality assessment

True positives, false positives, false negatives and true negatives were extracted from the publications or calculated from sensitivity, specificity and prevalence. If data were not available, we contacted authors by email.

The risk of bias and concerns regarding applicability of included articles was assessed by two independent reviewers (RC and RH) using the Quality Assessment for Diagnostic Accuracy Studies (QUADAS-2) tool.^15^ For this, RevMan V.5.3 software was used.^16^ Evidence quality was graded with the Grading of Recommendations Assessment, Development and Evaluation (GRADE) approach for diagnostic tests^17^ and evidence profile tables were created.

### Statistical analysis and data synthesis

**Figure 4:**
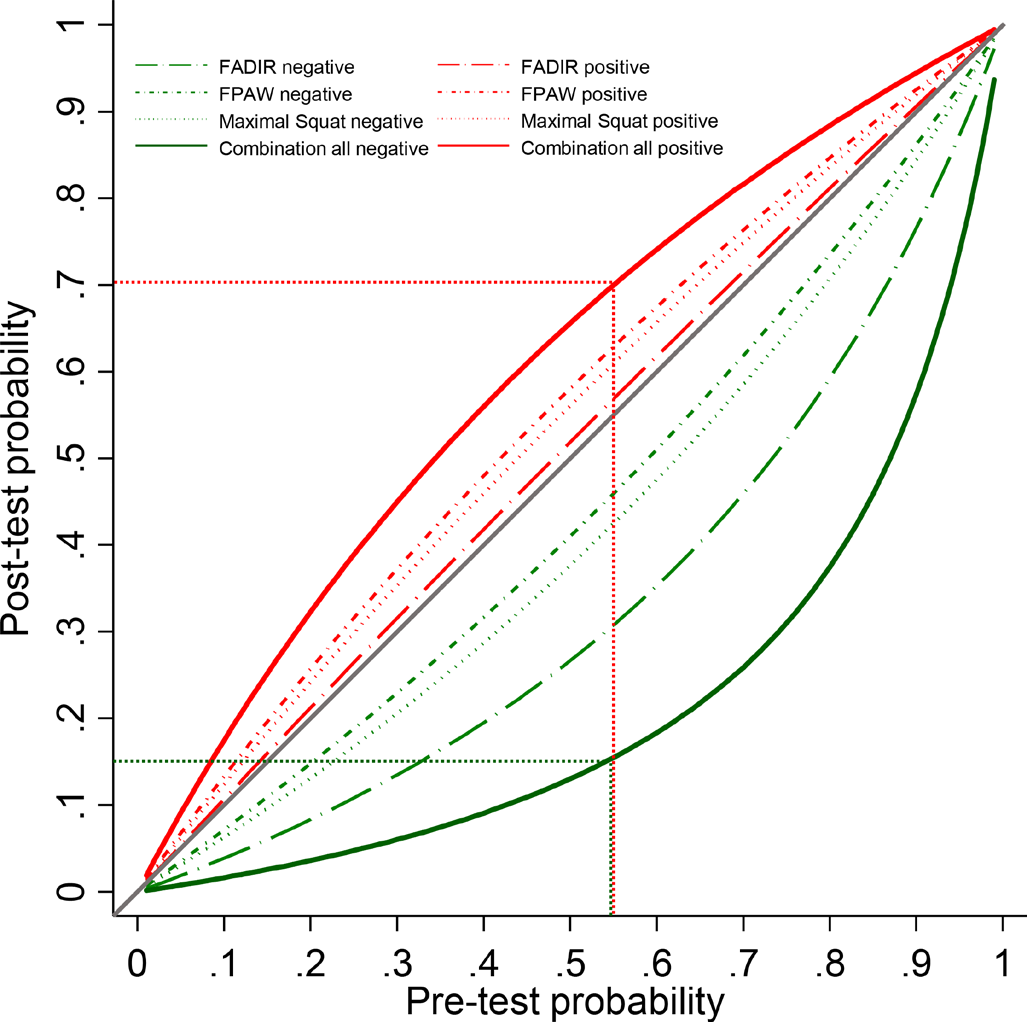
Post-test probabilities depending on varying prevalence (pretest probability). FADIR, flexion-adduction-internal rotation; FPAW, foot progression angle walking.

Diagnostic 2×2 tables were used to calculate sensitivity, specificity, disease prevalence and LRs for positive and negative test results (LR+, LR−). For each index test, we plotted the calculated sensitivities and specificities with their 95% CIs on forest plots for visual judgement of variation in test accuracy across studies. We planned to pool data if two or more studies reported data for the same test, but this did not occur. Positive and negative LRs were used to calculate post-test probabilities. An LR+ or LR− of 1 indicates no shift of the likelihood of disease, and values close to 1 indicate only small changes. The higher the LR+ above 1, the larger the increase of the probability of disease, while the lower the LR− below 1, the larger the decrease in the probability of the disease.^18^ To increase the changes from pretest to post-test probabilities (ie, to increase the diagnostic value), we combined several index tests by multiplying the corresponding LRs.^19^ Changes in post-test probabilities for the individual tests and for the test combination are presented using a plot showing the relationship between pretest and post-test probabilities for different disease prevalence (ie, pretest probabilities) (see figure 4).

### Amendments to the protocol

No meta-analysis could be performed because no test results with both sensitivity and specificity values were reported in more than one study. The execution and the positivity criteria of index tests were not precisely described in every included study, as required by the protocol. However, we did not exclude such manuscripts since this would have resulted in a small number of analysed studies. We did exclude studies that reported ultrasound as index test because ultrasound corresponds to imaging techniques and not to clinical tests. In a future study we plan to assess the diagnostic accuracy of ultrasound.

## Results

### Search results

**Figure 1:**
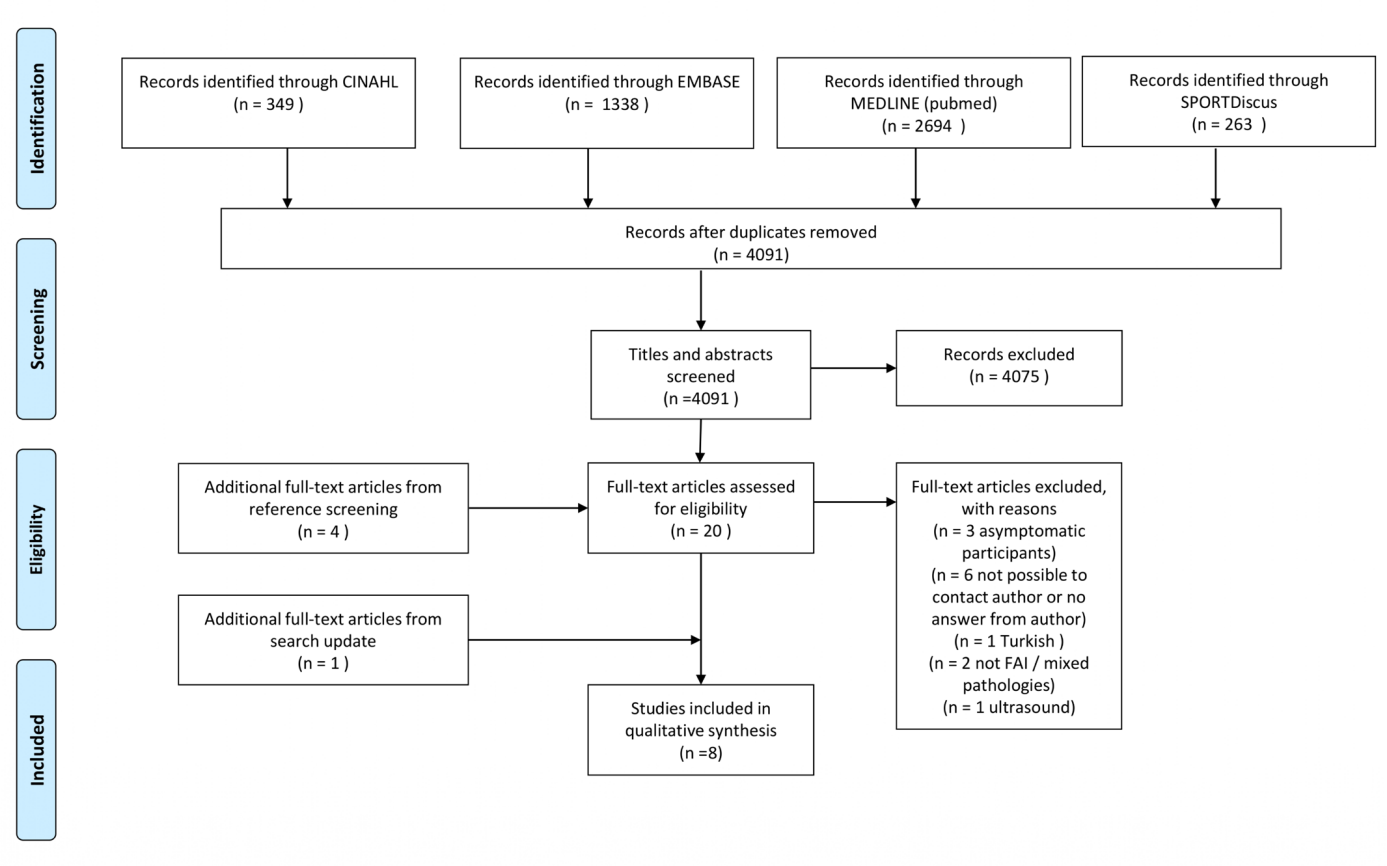
Study flow diagram. FAI, femoroacetabular impingement.

We screened 4091 titles and abstracts, and 21 full texts. Four studies were found by reference screening. Eight studies were included in this review. The study flow diagram, with reasons for exclusions, is presented in figure 1.

### Description of included studies

The characteristics of included studies are shown in the online supplementary table 1. The studies were performed in Canada,^20 21^ the USA^22–26^ and France.^27^ All studies were prospective. Seventeen clinical tests and two test combinations were reported. Three studies investigated the FABER test,^21–23^ but only one of these reported specificity.^23^ Two studies examined the FABER distance test^24 26^ (only one reported specificity).^26^ Two studies investigated the Stinchfield test^22 23^ (only one reported specificity).^23^ Two studies investigated the sensitivity of the posterior impingement test (PIT)^21 22^ (none reported specificity) and four studies investigated the FADIR test^21 22 24 25^ (only one reported specificity). For the remaining tests, there was only one study per test. In total, both sensitivity and specificity could be calculated for nine tests in five studies. For the other 10 clinical tests, only sensitivity could be calculated.

10.1136/bmjsem-2020-000772.supp1 Supplementary data

Studies provided data for 1666 hips. The prevalence of FAI morphology ranged from 10% to 64% (see table 1). Two studies^22 24^ included only participants with confirmed FAI syndrome, while from another study^21^ we extracted data from participants with confirmed FAI morphology and excluded asymptomatic participants so as to conform to our inclusion criteria.

**Table 1:** Overview of results and quality of evidence of tests with sensitivity and specificity data

Test name (author)	SN (95% CI)	SP (95% CI)	LR+ (95% CI)	LR− (95% CI)	Prevalence	Unit of analysis	QoE	
FPAW (Ranawat *et al*)^25^	0.61 (0.52 to 0.70)	0.56 (0.45 to 0.66)	1.386 (1.05 to 1.83)	0.696 (0.52 to 0.94)	0.558	Patients	SN	⨁⨁⨁◯ Moderate
					SP	⨁⨁⨁◯ Moderate
Maximal squat (Ayeni *et al*)^20^	0.75 (0.57 to 0.89)	0.41 (0.27 to 0.57)	1.278 (0.93 to 1.75)	0.605 (0.30 to 1.21)	0.41	Hips	SN	⨁⨁⨁◯ Moderate
					SP	⨁⨁⨁◯ Moderate
Pain predominantly in F/IR (Nogier *et al*)^27^	0.70 (0.62 to 0.77)	0.44 (0.33 to 0.55)	1.245 (1.01 to 1.54)	0.684 (0.49 to 0.96)	0.639	Patients	SN	⨁⨁◯◯ Low
					SP	⨁⨁◯◯ Low
FADIR (f90 add IR) (Ranawat *et al*)^25^	0.96 (0.91 to 0.99)	0.11 (0.06 to 0.20)	1.079 (0.99 to 1.17)	0.364 (0.12 to 1.08)	0.558	Patients	SN	⨁◯◯◯ Very low
					SP	⨁⨁◯◯ Low
FABER distance (Trindade *et al*)^26^	0.85 (0.79 to 0.90)	0.38 (0.33 to 0.42)	1.36 (1.23 to 1.50)	0.41 (0.28 to 0.59)	0.28	Patients	SN	⨁⨁◯◯ Low
					SP	⨁⨁⨁◯ Moderate
IROP (Maslowski *et al*)^23^	1 (0.48 to 1)	0.16 (0.06 to 0.29)	1.184 (0.83 to 1.44)	0 (0.03 to 7.87)	0.1	Patients	SN	⨁◯◯◯ Very low
					SP	⨁◯◯◯ Very low
Scour (Maslowski *et al*)^23^	0.8 (0.28 to 0.99)	0.40 (0.26 to 0.56)	1.333 (0.81 to 2.20)	0.5 (0.08 to 2.99)	0.1	Patients	SN	⨁◯◯◯ Very low
					SP	⨁◯◯◯ Very low
Stinchfield (RSLR)(Maslowski *et al*)^23^	0.6 (0.15 to 0.95)	0.36 (0.22 to 0.51)	0.931 (0.44 to 1.97)	1.125 (0.36 to 3.53)	0.1	Patients	SP	⨁◯◯◯ Very low
					SN	⨁◯◯◯ Very low
FABER (Maslowski *et al*)^23^	0.6 (0.15 to 0.95)	0.2 (0.10 to 0.35)	0.75 (0.36 to 1.56)	2 (0.59 to 6.79)	0.1	Patients	SN	⨁◯◯◯ Very low
					SP	⨁◯◯◯ Very low

FABER, flexion-abduction-external rotation; f90 add IR, flexion 90-adduction-internal rotation; FADIR, flexion-adduction-internal rotation; F/IR, flexion internal rotation; FPAW, foot progression angle walking; IROP, internal rotation over pressure; LR, likelihood ratio; QoE, Quality of Evidence; RSLR, resisted straight leg raise; SN, sensitivity; SP, specificity.

The reference tests used in the analysed studies were radiography,^21 22 24–27^ MRI/MRA^20^ or both radiography and MRI/MRA.^23^ Criteria for index and/or reference test positivity were not clearly stated in three out of eight studies.^21 23 25^

### Descriptions of included index tests

Fourteen of the analysed index tests were pain provocation tests: the maximal squat test,^20^ the FABER test,^21–23^ the log roll test,^22^ the resisted straight leg raise test (Stinchfield test),^22 23^ the FADIR test (anterior impingement test, flexion 90-adduction-internal rotation test (f90 add IR)),^21 22 24 25^ the PIT,^21 22^ the scour test,^23^ the internal rotation over pressure (IROP) test,^23^ the flexion plus IR pain test,^27^ the foot progression angle walking (FPAW) test,^25^ the IR pain test,^21^ the f120 add IR,^21^ the flexion 90-adduction-compression test (f90 add C)^21^ and the f120 add C.^21^ Five tests were range of motion (ROM) tests: two studies used the FABER distance test^24 26^ to compare the unaffected and affected hips in terms of loss of distance between the knee and examination table; there were also two passive hip ROM tests^21^ and two combinations of an ROM test plus a pain provocation test.

### Quality assessment and GRADE

**Figure 2:**
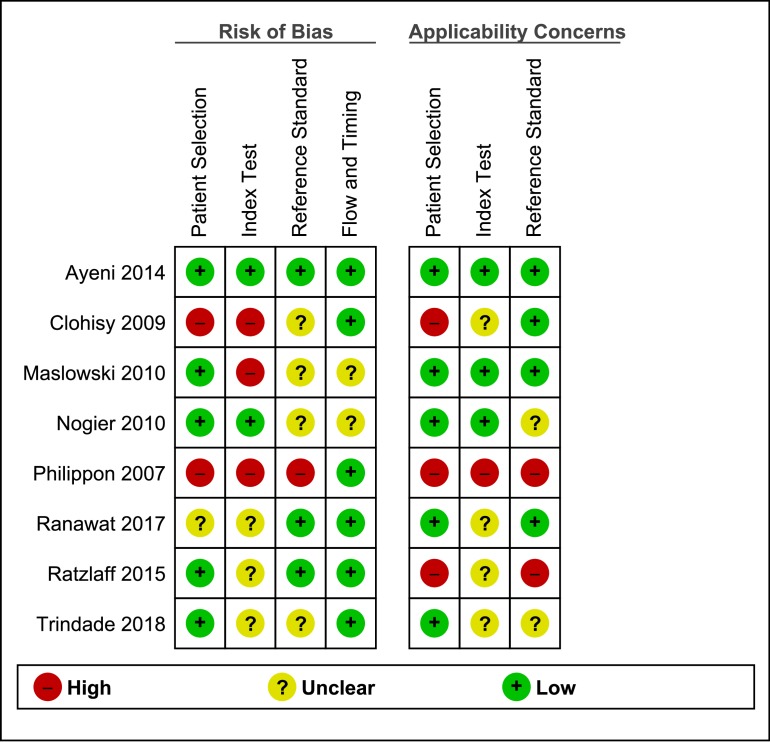
Results of the Quality Assessment for Diagnostic Accuracy Studies (QUADAS-2) tool.

Judgements of risks of bias and concerns regarding applicability were made using the QUADAS-2 tool, as shown in figure 2. Four studies showed a high risk of bias or concerns regarding applicability in at least one domain. Only one study^20^ was classified as having no risk of bias and no applicability concerns. In two studies,^22 24^ the investigators were informed before the testing that only participants with confirmed cam or pincer morphology were included. Ratings with GRADEpro demonstrated moderate-quality evidence for both the sensitivity and specificity of the FPAW test^25^ and the maximal squat test,^20^ as well as for the specificity of the FABER distance test.^26^ All other index tests showed low to very-low-quality evidence according to the GRADEpro rating. A short overview is presented in table 1; for the detailed grading of evidence see online supplementary file 5.

10.1136/bmjsem-2020-000772.supp5 Supplementary data

### Accuracy

**Figure 3:**
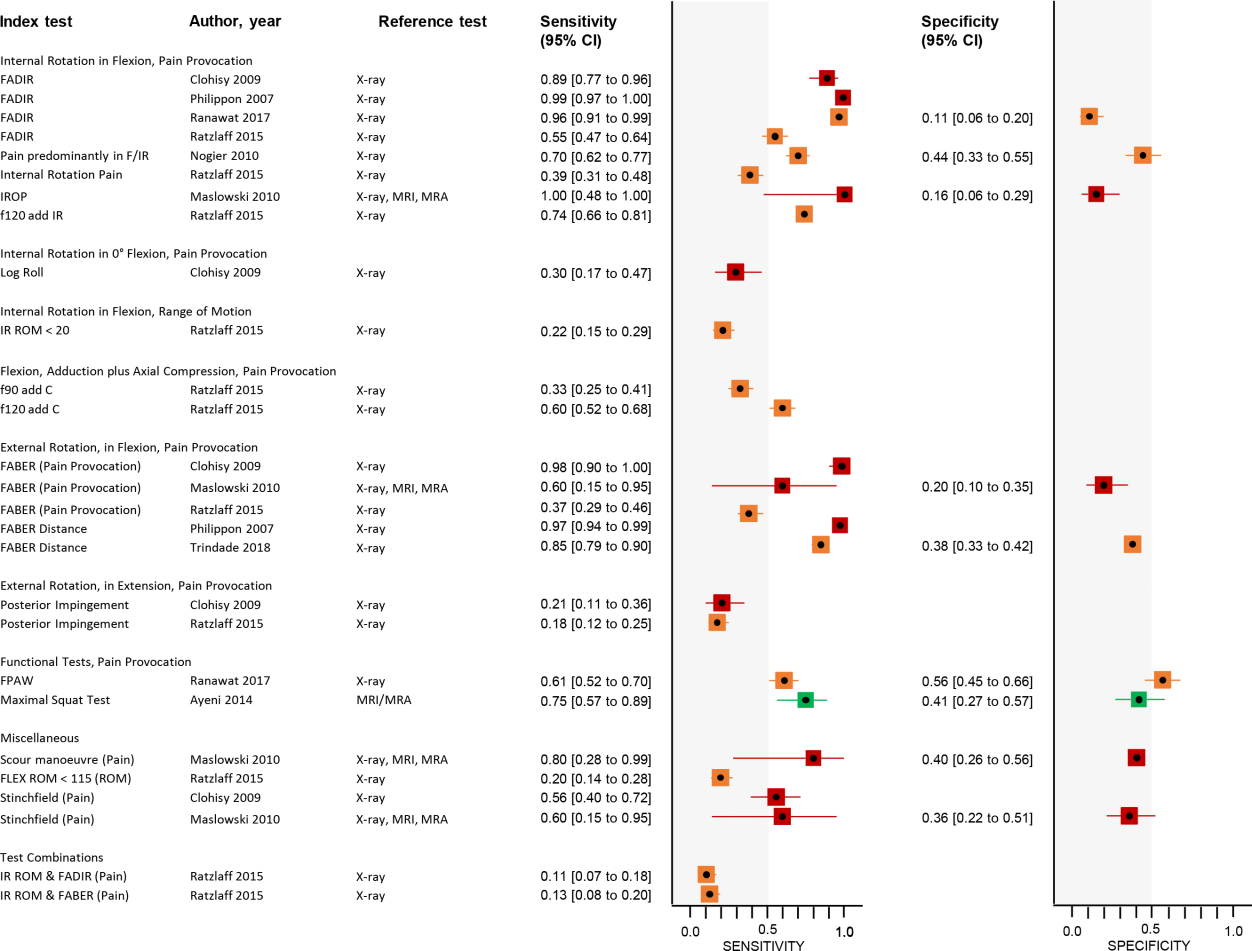
Forest plots of included clinical tests (see online supplementary file 4 for true positives, true negatives, false positives and false negatives). Red point estimates and CIs in the forest plot indicate high risk of bias (at least one item at high risk of bias), orange indicates unclear risk of bias (at least one unclear risk of bias and no high risk of bias), green indicates all risk of bias items at low risk. add, adduction; AIT, anterior impingement test; C, compression; f90, flexion 90°; f120, flexion 120°; FABER, flexion-abduction-external rotation; FADIR, flexion-adduction-internal rotation; FLEX, flexion; FPAW, foot progression angle walking; IR, internal rotation; IROP, internal rotation over pressure; MRA, magnetic resonance arthrography; ROM, range of motion; RSLR, resisted straight leg raise.

The test accuracy of each index test is presented using forest plots (figure 3). Sensitivity could be calculated for each of the index tests, and ranged from 0.11 (IR ROM and f90 IR, 95% CI 0.07 to 0.18) to 1.00 (IROP, 95% CI 0.48 to 1.00). The lowest sensitivity occurred in ROM tests, ranging from 0.11 to 0.22,^21^ and in PIT, ranging from 0.18 to 0.21.^21 22^

10.1136/bmjsem-2020-000772.supp4 Supplementary data

Specificity could be calculated for nine tests in five studies. The FPAW had the highest specificity at 0.56 (95% CI 0.45 to 0.66).^25^ The FADIR had the lowest specificity at 0.11 (95% CI 0.06 to 0.20).^25^

A summary of the accuracy data is shown in table 1.

### Likelihood ratios

The LRs of the nine tests were poor. No LR+ was higher than 1.4, and no LR− was smaller than 0.3. For two tests (the FABER and Stinchfield^23^), the LR+ and LR− pointed in the opposite direction, with an LR+ <1 and an LR− >1. The IROP test had a sensitivity of 100%, which results in an LR− of 0.00; however, the CI of the sensitivity ranged from 48% to 100%. None of the tests were able to relevantly shift the post-test probability. Figure 4 illustrates the small changes of post-test probabilities depending on the varying prevalence of cam or pincer morphology.

To increase the ability to rule out a cam or pincer morphology, we combined the three clinical tests with the highest possible sensitivity and smallest LR (ie, the FADIR, FPAW and maximal squat tests). Chaining of these tests resulted in an LR− of 0.15. In a population with a prevalence of 55%, the probability of a person having a cam or pincer morphology with three negative tests would decrease to 0.15 (figure 4). If the three tests were positive this would result in an LR+ of 1.91 and an increase of the post-test probability to 0.70 (figure 4).

## Discussion

This systematic review examined the current literature on clinical tests for the detection of cam or pincer morphology in individuals suspected of having FAI syndrome. Eight out of 4091 studies were included, and these reported on 17 clinical tests and two test combinations. Because of the insufficient number of studies per test, a meta-analysis could not be performed. There are three main findings: (1) there is only low-quality evidence; (2) no single test effectively rules in a cam or mixed morphology; (3) the FADIR, FPAW and the maximal squat test showed the best sensitivities and should be combined to cautiously rule out a cam or mixed morphology, but the validity of this combination should be tested with a multivariable regression model.

Specificity could only be calculated for nine tests. Overall results showed low specificity for all tests, ranging from 0.11 to 0.56. This indicates that these clinical tests might not be appropriate to rule in a cam or pincer morphology. High sensitivity was found for some pain provocation tests (the FADIR, FPAW, maximal squat) and for the FABER distance test. The interpretation of the FABER distance test, however, is questionable because the positivity criterion is a loss of distance between the lateral aspect of the knee and the examination table compared with the unaffected side. This requires the unaffected hip to be free of a cam or pincer morphology, but this can only be determined with imaging studies. Hence, this test is not applicable in physiotherapy practice. The lowest values for sensitivity were from a study for which we only had an abstract.^21^ No test reached a sensitivity above 0.75 in that study. Detailed information on test execution and criteria for a positive test were unavailable, and therefore findings from that study should be interpreted with caution.

LRs could be calculated for nine tests. The LRs only allow for small changes from pretest to post-test probabilities. However, the combination of three negative test results in the FADIR, FPAW and maximal squat tests yielded an LR− of 0.15. Unexpected results were obtained for the Stinchfield and FABER tests, where the LR+ was below 1 and the LR− above 1. These two tests were investigated in the same study,^23^ with a prevalence of FAI morphology of 10%. All subjects were suspected to have intra-articular pathology, which might explain the high false positive rate.

A higher suspicion of cam and pincer morphology may result in a higher sensitivity of clinical tests, because evaluators might rate the test as positive in cases where the result is less clear; this would in turn decrease the number of false negatives. Three studies^21 22 24^ included only patients with confirmed FAI deformities. In two studies,^22 24^ the raters were aware of this fact, while in one study the manuscript was unclear regarding the blinding status.

The reference test in most of the included studies was radiography, though one study^23^ used three different imaging techniques (MRI, MRA, X-ray) and one^20^ used MRI/MRA. Further, the included studies showed varying criteria for positivity of the reference test. They defined different alpha angle values for the diagnosis of cam morphology, as well as varying positivity criteria for pincer morphology, making them difficult to compare. Of the three tests proposed for our test combination, the maximal squat test was compared with MRI/MRA (head-neck offset <9 mm or alpha angle >55°), while the FPAW and the FADIR were compared with radiography (alpha angle >60°).

It is known that cam or pincer morphology can lead to labral and cartilage damage. Both types of damage are considered to be risk factors for early degenerative processes and osteoarthritis of the hip joint, due to reduced hip joint motions, elevated contact pressures and shear stress caused by cam and pincer deformities.^3 28–31^ It is important to recognise that lesions of the labrum can occur as a consequence of impingement but are not present in all cases.^32^ Thus, studies including participants who have only labral lesions are not appropriate for assessing the accuracy of tests for cam or pincer morphology. There is an association between cam morphology and the development of osteoarthritis, whereas pincer morphology (in contrast to acetabular dysplasia) does not seem to be a risk factor for osteoarthritis.^31 33^

It is not possible to make a general statement on whether sensitivity or specificity is more important. This depends on the context in which we apply a test. In the context of professional athletes, the sensitivity of a test should be high, so as not to miss potential cam or pincer morphologies. A diagnosis of such morphologies will have consequences on the athlete’s training or competing behaviour and might even have an impact on the pursuit of his career. In contrast, in a general population screening process we want the specificity to be high, so as not to have too many false positives, the impact of missing one case in that population is less serious.

Strengths of this systematic review include the facts that wherever possible, we clearly stated the types of clinical tests investigated—ROM, pain provocation or imaging—and precisely described the positivity criteria. We considered only studies that included symptomatic participants. This was done to meet the official definition of FAI syndrome, where symptoms are mandatory and asymptomatic individuals are not diagnosed with this condition.^12^ The guidelines of the Cochrane Handbook for Systematic Reviews of Diagnostic Test Accuracy and the PRISMA-DTA checklist were followed to ensure sound scientific practice. Data extraction, estimation of risk of bias and grading of evidence were performed by two independent reviewers. The findings of this review were presented visually in forest plots to provide a simple, quick and informative overview of the test accuracy of clinical tests. Furthermore, a test combination was designed to help practitioners apply the findings to clinical practice. Figure 4 permits the quick identification of post-test probabilities for different prevalences and tests. In comparison to a review published by Reiman *et al*,^10^ this report has several advantages. First, data of two additional studies were analysed. Second, only studies with symptomatic participants were included. Third, FAI deformities were clearly differentiated from labral tears alone.

A limitation of this review is that the proposition of chaining clinical tests might result in an overestimation of the post-test probability, if the combined tests are not fully independent.^34^ The value of this test combination should be evaluated in a new study with a multivariable logistic regression model. Additionally, three^21 22 24^ out of eight studies included only cases and hence, there was no clinical uncertainty, which introduces high risk of bias.

There were several limitations of the included studies. Most of the studies had a high risk of bias and rather low statistical precision. Different diagnostic criteria were used for the radiographic definition of cam or pincer morphology, as mentioned above, and in some cases, there was no clear statement of the diagnostic criteria. A further limitation is that the diagnostic test accuracy was not reported separately for cam, pincer or mixed morphology. In our proposed test combination, we included one test (maximal squat) from a study that diagnosed cam morphology, and two tests (the FADIR and FPAW) from a study that diagnosed FAI, defined as cam, pincer or mixed morphology. The inclusion of patients with only pincer morphology would probably lower the diagnostic test accuracy of the tests (see ref 35). Therefore, our suggestion is valid for the detection of cam or mixed morphologies. We cannot make a recommendation for the detection of pure pincer morphology.

There is a need for studies with larger numbers of participants, clear definitions of the diagnostic criteria of the reference tests and clear distinctions between patient subgroups (ie, those with cam morphology only, pincer morphology only or mixed-type morphologies) and between those with or without labral tears. Symptomatic patients with acetabular labral tears alone should not be considered as having FAI syndrome. Future studies should always include cases and non-cases so that sensitivity and specificity can be calculated, and the risk of bias should be reduced, especially by blinding the assessors concerning the patient’s morphology.

There is only low-quality evidence that negative test results reduce the post-test probability of cam or mixed morphologies to a moderate amount and that consecutive testing with the FADIR, FPAW and maximal squat tests might be used as a clinical test combination. Due to the low specificity of clinical tests, we would not recommend their use to confirm the diagnosis of FAI syndrome. But so far, we do not have strong information about the interpretability of these test results, that is, there is too high uncertainty due to low-quality evidence and high risk of bias. Therefore, further adequately designed studies in larger populations and with different patient settings are required.
