# Supplementary material for: Diagnostic accuracy of clinical tests for cam or pincer morphology in individuals with suspected FAI syndrome: a systematic review
Source: BMJ Open Sport Exerc Med. 2020 Apr 27;6(1):e000772. doi: 10.1136/bmjsem-2020-000772 (PMC7213881; doi:10.1136/bmjsem-2020-000772)
Supplement: Supplementary data [file bmjsem-2020-000772supp006.pdf]

Search strategy for MEDLINE Database (via PubMed)

((((((((((((((((((((((((((MRI) OR MRA) OR MRT) OR arthroscopy) OR arthroscopic) OR radiography) OR radiographic) OR CT) OR ultrasound) OR surgery OR fluoroscopic))

AND

(((((((((((((((((((((((((((physical examination) OR clinical examination) OR clinical test) OR clinical evaluation) OR musculoskeletal evaluation) OR musculoskeletal evaluation) OR AIT) OR anterior impingement test) OR posterior impingement test) OR FABER) OR flexion abduction external rotation) OR Fitzgerald test) OR FADDIR) OR FADIR) OR flexion adduction internal rotation) OR scour) OR IROP) OR internal rotation overpressure) OR RSLR) OR stinchfield) OR maximal squat test) OR Bilateral lower extremity squat) OR Thomas test) OR Flexion Internal rotation) OR Internal rotation Flexion Compression) OR THIRD) OR the hip internal rotation with distraction OR provocation))

AND

((((((((((((femoroacetabular impingement) OR femoro-acetabular impingement) OR hip impingement) OR FAI) OR femoroacetabular syndrome) OR femoro-acetabular syndrome) OR cam) OR pincer) OR labral tear) OR labral tears) OR acetabular tear) OR acetabular tears OR (intra articular AND hip))))

NOT

shoulder[Title]) NOT glenoid[Title]) NOT dental[Title]) NOT mandible[Title]) NOT mandibular[Title]) NOT fascial[Title]) NOT dementia[Title]) NOT alzheimer[Title]) NOT oncologic[Title]) NOT cancer[Title]) NOT oncological[Title]) NOT cardio[Title]) NOT cardiac[Title]
