# Supplementary material for: Diagnostic accuracy of clinical tests for cam or pincer morphology in individuals with suspected FAI syndrome: a systematic review
Source: BMJ Open Sport Exerc Med. 2020 Apr 27;6(1):e000772. doi: 10.1136/bmjsem-2020-000772 (PMC7213881; doi:10.1136/bmjsem-2020-000772)
Supplement: Supplementary data [file bmjsem-2020-000772supp002.pdf]

Table 3S diagnostic accuracy of different imaging index tests used in included studies

Imaging Index Test		Reference Test	Sensitivity	Specificity	Reference
MRA	versus	Arthroscopy	0.79	1	González 2015 (1)
MRA	versus	Arthroscopy	0.34	0.82	Rajeev 2018 (2)
MRA	versus	Arthroscopy	1	1	Aprato 2013 (3)
MRA	versus	Surgical dislocation	1	1	Aprato 2013 (3)
x-Ray AP	versus	MRI	0.6	0.81	Barton 2011 (4)
x-Ray cross table	versus	MRI	0.74	0.63	Barton 2011(4)
x-Ray Dunn	versus	MRI	0.91	0.88	Barton 2011 (4)

1 González Gil AB, Llombart Blanco R, Díaz de Rada P. Validity of magnetic resonance arthrography as a diagnostic tool in femoroacetabular impingement syndrome. *Revista Española de Cirugía Ortopédica y Traumatología (English Edition)* 2015;4:281-6 doi:10.1016/j.recote.2015.04.009.
2 Rajeev A, Tuinebreijer W, Mohamed A, Newby M. The validity and accuracy of MRI arthrogram in the assessment of painful articular disorders of the hip. *Eur J Orthop Surg Traumatol* 2018;1:71-7 doi:10.1007/s00590-017-2022-9.
3 Aprato A, Masse A, Faletti C, Valente A, Atzori F, Stratta M, et al. Magnetic resonance arthrography for femoroacetabular impingement surgery: is it reliable? *J Orthop Traumatol* 2013;3:201-6 doi:10.1007/s10195-013-0227-1.
4 Barton C, Salineros MJ, Rakhra KS, Beaule PE. Validity of the alpha angle measurement on plain radiographs in the evaluation of cam-type femoroacetabular impingement. *Clin Orthop Relat Res* 2011;2:464-9 doi:10.1007/s11999-010-1624-x.
