# Supplementary material for: Diagnostic accuracy of clinical tests for cam or pincer morphology in individuals with suspected FAI syndrome: a systematic review
Source: BMJ Open Sport Exerc Med. 2020 Apr 27;6(1):e000772. doi: 10.1136/bmjsem-2020-000772 (PMC7213881; doi:10.1136/bmjsem-2020-000772)
Supplement: Supplementary data [file bmjsem-2020-000772supp003.pdf]

Table 4S reliability of plain radiography for measuring alpha angles (1)

	Intraobserver reliability (with CIs)	Interobserver reliability (with CIs)
AP view on X-ray	0.884 (0.746–0.949)	0.947 (0.909–0.969)
Cross table view on X-ray	0.947 (0.88–0.977)	0.85 (0.751–0.911)
Dunn view on X-ray	0.979 (0.952–0.991)	0.90 (0.832–0.942

1 Barton C, Salineros MJ, Rakhra KS, Beaule PE. Validity of the alpha angle measurement on plain radiographs in the evaluation of cam-type femoroacetabular impingement. *Clin Orthop Relat Res* 2011;2:464-9 doi:10.1007/s11999-010-1624-x.
