# Supplementary material for: Diagnostic accuracy of clinical tests for cam or pincer morphology in individuals with suspected FAI syndrome: a systematic review
Source: BMJ Open Sport Exerc Med. 2020 Apr 27;6(1):e000772. doi: 10.1136/bmjsem-2020-000772 (PMC7213881; doi:10.1136/bmjsem-2020-000772)
Supplement: Supplementary data [file bmjsem-2020-000772supp001.pdf]

Table 1 Characteristics of included studies

Author	Year	Country	Study design	Study aim	Setting	Types of participants	Sample size	Age (y)	Sex	Clinical tests	Criteria for positive testing	Reference test	Criteria for positive reference standard
**Ayeni et al.**	**2014**	Canada	prospective cohort study	to determine the sensitivity and specificity of a maximal Squat test for a cam deformity	adult outpatient orthopaedic clinic	adults with hip pain, an MRI/MRA of the affected hip and ability to perform an unassisted maximal squat	78 hips	mean 38.3	37 males, 39 females	maximal Squat	recreation of typical hip and groin pain while squatting	MRI / MRA: axial oblique sequences	cam: alpha angle > 55° and/or femoral head-neck offset < 9 mm
**Clohisy et al.**	**2009**	USA	prospective cohort study	to describe clinical history, function and physical examination findings associated with symptomatic FAI	not stated	patients with confirmed symptomatic FAI (by X-ray), scheduled for surgery	51 patients[1]	mean 35	29 males, 22 females	FABER<br>log roll<br>Stinchfield (=RSLR)<br>FADIR (=AIT)<br>posterior impingement test (PIT)	groin pain	X-ray: a/p, frog leg lateral, cross-table lateral	cam: aspherical femoral head, femoral head-neck offset < 9 mm<br>pincer: acetabular retroversion, coxa profunda, coxa protrusio
**Maslowski et al.**	**2010**	USA	prospective diagnostic validity study	to validate the diagnostic utility of hip provocation maneuvers to predict the presence of intra-articular hip pathology	multispecialty musculoskeletal clinic at a university medical center	subjects ≥ 18y, referred for injection with typical symptoms, physical examination findings and radiography suggesting intra-articular hip pain	50 patients	mean 60	20 males, 30 females	FABER<br>Stinchfield (=RSLR)<br>Scour<br>IROP	recreation of typical hip pain<br>recreation of typical hip pain<br>recreation of hip pain<br>recreation of hip pain	X-ray, MRI, MRA	not stated

Author	Year	Country	Study design	Study aim	Setting	Types of participants	Sample size	Age (y)	Sex	Clinical tests	Criteria for positive testing	Reference test	Criteria for positive reference standard
**Nogier et al.**	**2010**	France	prospective multicenter study	to assess the prevalence of dysplasia and femoroacetabular impingement	four surgical centers	mechanical hip pathology, pain more than 4 months	241 patients	16-50	292 participants, 62% males, 38% females	flexion plus internal rotation	pain, predominantly during flexion/internal rotation	X-ray: a/p, Lequesne false profile, lateral axial (Ducro-quet or Dunn lateral view)	cam: femoral head bump, anterosuperior neck flatness or ovoid head (on AP or lateral axial view)<br>pincer: crossover sign or acetabular protrusion
**Philippon et al.**	**2007**	USA	prospective cohort study	to identify subjective complaints and objective findings in patients treated for FAI	patients treated by a senior surgeon	patients treated for FAI: osteoplasty of femoral neck and / or acetabular rim trimming	301 patients	mean 40	153 males, 148 females	FADIR (=AIT)<br>FDT	hip pain<br>any loss of distance between the lateral aspect of the knee and the examination table, compared to the unaffected hip	X-ray: a/p, cross-table lateral view	cam: decreased offset of superior or anterior femoral head-neck junction<br>pincer: coxa profunda, acetabular protrusion or retroversion, cross-over sign
**Ranawat et al.**	**2017**	USA New York	prospective study	to evaluate the Foot Progression Angle Walking (FPAW) test as a diagnostic tool for FAI and hip instability	hospital	subjects with hip pain	199 patients	18-65	85 males, 114 females	FPAW<br>FADIR (=AIT)	presence of hip pain during testing or exacerbation of symptoms if pain was present at baseline<br>not stated	X-ray: a/p, elongated neck lateral view	cam or mixed form: alpha angle ≥ 60°,<br>pincer: center-edge angle > 30°
**Ratzlaff et al.**	**2015**	Canada	prospective cohort study, only	to estimate the prevalence and diagnostic	university	Caucasian people with FAI and pain	143 hips	20-49	510 Caucasian participants,	FABER<br>Internal rotation pain	not stated	X-ray: a/p,	FAI: any one of 1) lateral center edge

Author	Year	Country	Study design	Study aim	Setting	Types of participants	Sample size	Age (y)	Sex	Clinical tests	Criteria for positive testing	Reference test	Criteria for positive reference standard
			abstract, no full text	accuracy of physical exam tests in identifying radiographic FAI					35.7% male, 64.3% female	post impingement (PIT)<br>f120 add IR<br>f90 add IR (FADIR)<br>f90 add C<br>f120 add C<br>IR ROM <20<br>FLEX ROM <115<br>IR ROM & FABER<br>IR ROM & f90 IR		bilateral Dunn	angle (LCE) > 40°, 2) alpha angle > 55° and 3) presence of a cross-over sign
**Trindade et al.**	**2018**	USA	prospective cohort study	to determine if the FABER distance test (FDT) was associated with the alpha angle as a diagnostic tool for FAI	not stated	patients with symptomatic unilateral FAI who underwent hip arthroscopy. Patients were included if they had hip pain, alpha angle ≥ 50°, ≥ 18 years, prospectively documented FDT and alpha angle and complete physical exam	603 patients	18-71, 36.4 mean	344 males, 259 female	FDT	distance from the lateral femoral epicondyle of the knee to the examination table, with a difference between sides greater than 4 cm	X-ray: Cross-table	alpha angle ≥ 78°

[1] varying number of hips tested for each clinical test

Author	Year	Country	Study design	Study aim	Setting	Types of participants	Sample size	Age (y)	Sex	Clinical tests	Criteria for positive testing	Reference test	Criteria for positive reference standard
AIT: Anterior Impingement Test, add: Adduction, C: Compression, f90: Flexion 90°, f120: Flexion 120°, FABER: Flexion Abduction External Rotation, FADIR: Flexion Adduction Internal Rotation, FAI: Femoroacetabular Impingement, FDT: FABER Distance Test, FPAW: Foot Progression Angle Walking, IROP: Internal Rotation Over Pressure, IR: Internal Rotation, MRI: Magnetic Resonance Imaging, MRA: Magnetic Resonance Arthrography, PIT: Posterior Impingement Test, ROM: Range Of Motion, RSLR: Resisted Straight Leg Raise, X-ray: Radiography													
