# Supplementary material for: Diagnostic accuracy of clinical tests for cam or pincer morphology in individuals with suspected FAI syndrome: a systematic review
Source: BMJ Open Sport Exerc Med. 2020 Apr 27;6(1):e000772. doi: 10.1136/bmjsem-2020-000772 (PMC7213881; doi:10.1136/bmjsem-2020-000772)
Supplement: Supplementary data [file bmjsem-2020-000772supp005.pdf]

2 GRADEpro evidence profile tables for all index tests

Judgement of the evidence quality with GRADEpro was made according to the following criteria:

Risk of bias:

not serious: if all risk of bias items were at low risk according to the QUADAS 2 tool

serious: if at least one item was at unclear risk and no high risk according to the QUADAS 2 tool

very serious: if at least one item was at high risk according to the QUADAS 2 tool

For detailed information of risk of bias rating, please check figure 3 (the QUADAS 2 tool).

Indirectness: Where positivity of the index or reference test or the execution of the index test were not clearly mentioned in the study, indirectness was rated as serious.

Inconsistency: If there was an unexplained heterogeneity of results across studies, inconsistency was rated as serious.

Based on the partially low prevalences in the included studies we decided a number of participants less than 250 as a serious imprecision.

Question: Should f120 add C be used to diagnose pincer or cam deformities in FAI syndrome?

Sensitivity	0.60 (95% CI: 0.52 to 0.68)
Specificity	-- (95% CI: -- to --)

Prevalences	55%	85%	

Outcome	№ of studies (№ of patients)	Study design	Factors that may decrease certainty of evidence					Effect per 1.000 patients tested		Test accuracy CoE
			Risk of bias	Indirectness	Inconsistency	Imprecision	Publication bias	pre-test probability of 55%	pre-test probability of 85%	
**True positives** (patients with pincer or cam deformities)	1 studies 143 patients	case-control type accuracy study	very serious [a]	serious [b]	not serious	serious [c]	none	330 (286 to 374)	510 (442 to 578)	⊕◯◯◯ VERY LOW
**False negatives** (patients incorrectly classified as not having pincer or cam deformities)								220 (176 to 264)	340 (272 to 408)	
**True negatives** (patients without pincer or cam deformities)	0 studies 0 patients							0 (0 to 0)	0 (0 to 0)	-
**False positives** (patients incorrectly classified as having pincer or cam deformities)								450 (450 to 450)	150 (150 to 150)	

Explanations

a. Inclusion criteria do not exactly match the review question, execution and interpretation of index and reference test not described
b. Test execution and interpretation not described
c. small number of participants

Question: Should f120 add IR be used to diagnose pincer or cam deformities in FAI syndrome?

Sensitivity	0.74 (95% CI: 0.66 to 0.81)
Specificity	-- (95% CI: -- to --)

Prevalences	55%	85%	

Outcome	№ of studies (№ of patients)	Study design	Factors that may decrease certainty of evidence					Effect per 1.000 patients tested		Test accuracy CoE
			Risk of bias	Indirectness	Inconsistency	Imprecision	Publication bias	pre-test probability of 55%	pre-test probability of 85%	
**True positives** (patients with pincer or cam deformities)	1 studies 143 patients	case-control type accuracy study	very serious [a]	serious [b]	not serious	serious [c]	none	407 (363 to 446)	629 (561 to 689)	⨁◯◯◯ VERY LOW
**False negatives** (patients incorrectly classified as not having pincer or cam deformities)								143 (104 to 187)	221 (161 to 289)	
**True negatives** (patients without pincer or cam deformities)	0 studies 0 patients	cross-sectional (cohort type accuracy study)						0 (0 to 0)	0 (0 to 0)	-
**False positives** (patients incorrectly classified as having pincer or cam deformities)								450 (450 to 450)	150 (150 to 150)	

Explanations

a. Inclusion criteria do not exactly match the review question, execution and interpretation of index and reference test not described
b. Test execution and interpretation not described
c. small number of participants

Question: Should f90 add C be used to diagnose pincer or cam deformities in FAI syndrome?

Sensitivity	0.33 (95% CI: 0.25 to 0.41)
Specificity	-- (95% CI: -- to --)

Prevalences	55%	85%	

Outcome	№ of studies (№ of patients)	Study design	Factors that may decrease certainty of evidence					Effect per 1.000 patients tested		Test accuracy CoE
			Risk of bias	Indirectness	Inconsistency	Imprecision	Publication bias	pre-test probability of 55%	pre-test probability of 85%	
**True positives** (patients with pincer or cam deformities)	1 studies 143 patients	case-control type accuracy study	very serious [a]	serious [b]	not serious	serious [c]	none	182 (138 to 226)	281 (213 to 349)	⨁◯◯◯ VERY LOW
**False negatives** (patients incorrectly classified as not having pincer or cam deformities)								368 (324 to 412)	569 (501 to 637)	
**True negatives** (patients without pincer or cam deformities)	0 studies 0 patients	cross-sectional (cohort type accuracy study)						0 (0 to 0)	0 (0 to 0)	-
**False positives** (patients incorrectly classified as having pincer or cam deformities)								450 (450 to 450)	150 (150 to 150)	

Explanations

a. Inclusion criteria do not exactly match the review question, execution and interpretation of index and reference test not described
b. Test execution and interpretation not described
c. small number of participants

Question: Should FABER Distance test (FDT) be used to diagnose pincer or cam deformities in FAI syndrome?

Sensitivity	0.79 to 0.99
Specificity	0.33 to 0.42

Prevalences	55%	85%	

Outcome	№ of studies (№ of patients)	Study design	Factors that may decrease certainty of evidence					Effect per 1.000 patients tested		Test accuracy CoE
			Risk of bias	Indirectness	Inconsistency	Imprecision	Publication bias	pre-test probability of 55%	pre-test probability of 85%	
**True positives** (patients with pincer or cam deformities)	2 studies 472 patients	cohort & case-control type studies	very serious [a]	not serious	not serious	not serious	none	435 to 545	672 to 842	⨁⨁◯◯ LOW
**False negatives** (patients incorrectly classified as not having pincer or cam deformities)								5 to 115	8 to 178	
**True negatives** (patients without pincer or cam deformities)	1 studies 432 patients	cross-sectional (cohort type accuracy study)	serious [a]	not serious	not serious	not serious	none	149 to 189	50 to 63	⨁⨁⨁◯ MODERATE
**False positives** (patients incorrectly classified as having pincer or cam deformities)								261 to 301	87 to 100	

Explanations

a. see QUADAS 2

Question: Should FABER test (Patrick) be used to diagnose pincer or cam deformities in FAI syndrome?

Sensitivity	0.15 to 1.00
Specificity	0.10 to 0.35

Prevalences	55%	85%	

Outcome	№ of studies (№ of patients)	Study design	Factors that may decrease certainty of evidence					Effect per 1.000 patients tested		Test accuracy CoE
			Risk of bias	Indirectness	Inconsistency	Imprecision	Publication bias	pre-test probability of 55%	pre-test probability of 85%	
**True positives** (patients with pincer or cam deformities)	3 studies 201 patients	cohort & case-control type studies	very serious [a]	serious [b]	serious [c]	serious [d]	none	83 to 550	128 to 850	⨁◯◯◯ VERY LOW
**False negatives** (patients incorrectly classified as not having pincer or cam deformities)								0 to 467	0 to 722	
**True negatives** (patients without pincer or cam deformities)	1 studies 45 patients	cross-sectional (cohort type accuracy study)	very serious [a]	not serious	not serious	serious [d]	none	45 to 157	15 to 53	⨁◯◯◯ VERY LOW
**False positives** (patients incorrectly classified as having pincer or cam deformities)								293 to 405	97 to 135	

Explanations

a. see QUADAS 2
b. reference test positivity not always stated
c. wide variance of point estimates across studies
d. small number of participants

Question: Should FADIR test (AIT, f90 add IR) be used to diagnose pincer or cam deformities in FAI syndrome?

Sensitivity	0.47 to 1.00
Specificity	0.06 to 0.20

Prevalences	55%	85%	

Outcome	№ of studies (№ of patients)	Study design	Factors that may decrease certainty of evidence					Effect per 1.000 patients tested		Test accuracy CoE
			Risk of bias	Indirectness	Inconsistency	Imprecision	Publication bias	pre-test probability of 55%	pre-test probability of 85%	
**True positives** (patients with pincer or cam deformities)	4 studies 608 patients	cohort & case-control type studies	very serious [a]	not serious	serious [b]	not serious	none	259 to 550	399 to 850	⨁◯◯◯ VERY LOW
**False negatives** (patients incorrectly classified as not having pincer or cam deformities)								0 to 291	0 to 451	
**True negatives** (patients without pincer or cam deformities)	1 studies 88 patients	cross-sectional (cohort type accuracy study)	serious [a]	not serious	not serious	serious [c]	none	27 to 90	9 to 30	⨁⨁◯◯ LOW
**False positives** (patients incorrectly classified as having pincer or cam deformities)								360 to 423	120 to 141	

Explanations

a. see QUADAS 2
b. wide variance of point estimates across studies
c. small number of participants

Question: Should FLEX ROM < 115 be used to diagnose pincer or cam deformities in FAI syndrome?

Sensitivity	0.20 (95% CI: 0.14 to 0.28)
Specificity	-- (95% CI: -- to --)

Prevalences	55%	85%	

Outcome	№ of studies (№ of patients)	Study design	Factors that may decrease certainty of evidence					Effect per 1.000 patients tested		Test accuracy CoE
			Risk of bias	Indirectness	Inconsistency	Imprecision	Publication bias	pre-test probability of 55%	pre-test probability of 85%	
**True positives** (patients with pincer or cam deformities)	1 studies 143 patients	case-control type accuracy study	very serious [a]	serious [b]	not serious	serious [c]	none	110 (77 to 154)	170 (119 to 238)	⊕○○○ VERY LOW
**False negatives** (patients incorrectly classified as not having pincer or cam deformities)								440 (396 to 473)	680 (612 to 731)	
**True negatives** (patients without pincer or cam deformities)	0 studies 0 patients							0 (0 to 0)	0 (0 to 0)	-
**False positives** (patients incorrectly classified as having pincer or cam deformities)								450 (450 to 450)	150 (150 to 150)	

Explanations

a. Inclusion criteria do not exactly match the review question, execution and interpretation of index and reference test not described
b. Test execution and interpretation not described
c. small number of participants

Question: Should FPAW be used to diagnose pincer or cam deformities in FAI syndrome?

Sensitivity	0.61 (95% CI: 0.52 to 0.70)
Specificity	0.56 (95% CI: 0.45 to 0.66)

Prevalences	55%	85%	

Outcome	№ of studies (№ of patients)	Study design	Factors that may decrease certainty of evidence					Effect per 1.000 patients tested		Test accuracy CoE
			Risk of bias	Indirectness	Inconsistency	Imprecision	Publication bias	pre-test probability of 55%	pre-test probability of 85%	
**True positives** (patients with pincer or cam deformities)	1 studies 111 patients	cross-sectional (cohort type accuracy study)	not serious	not serious	not serious	serious [a]	none	336 (286 to 385)	519 (442 to 595)	⨁⨁⨁◯ MODERATE
**False negatives** (patients incorrectly classified as not having pincer or cam deformities)								214 (165 to 264)	331 (255 to 408)	
**True negatives** (patients without pincer or cam deformities)	1 studies 88 patients	cross-sectional (cohort type accuracy study)	not serious	not serious	not serious	serious [a]	none	252 (202 to 297)	84 (68 to 99)	⨁⨁⨁◯ MODERATE
**False positives** (patients incorrectly classified as having pincer or cam deformities)								198 (153 to 248)	66 (51 to 82)	

Explanations

a. small number of participants

Question: Should Internal Rotation Pain test be used to diagnose pincer or cam deformities in FAI syndrome?

Sensitivity	0.39 (95% CI: 0.31 to 0.48)
Specificity	-- (95% CI: -- to --)

Prevalences	55%	85%	

Outcome	№ of studies (№ of patients)	Study design	Factors that may decrease certainty of evidence					Effect per 1.000 patients tested		Test accuracy CoE
			Risk of bias	Indirectness	Inconsistency	Imprecision	Publication bias	pre-test probability of 55%	pre-test probability of 85%	
**True positives** (patients with pincer or cam deformities)	1 studies 143 patients	case-control type accuracy study	very serious [a]	serious [b]	not serious	serious [c]	none	215 (171 to 264)	332 (264 to 408)	⨁◯◯◯ VERY LOW
**False negatives** (patients incorrectly classified as not having pincer or cam deformities)								335 (286 to 379)	518 (442 to 586)	
**True negatives** (patients without pincer or cam deformities)	0 studies 0 patients	cross-sectional (cohort type accuracy study)						0 (0 to 0)	0 (0 to 0)	-
**False positives** (patients incorrectly classified as having pincer or cam deformities)								450 (450 to 450)	150 (150 to 150)	

Explanations

a. inclusioncriteria do not exactly match the review question, execution and interpretation of index and reference test not described
b. Test execution and interpretation not described
c. small number of participants

Question: Should IR ROM & f90 IR be used to diagnose pincer or cam deformities in FAI syndrome?

Sensitivity	0.11 (95% CI: 0.07 to 0.18)
Specificity	-- (95% CI: -- to --)

Prevalences	55%	85%	

Outcome	№ of studies (№ of patients)	Study design	Factors that may decrease certainty of evidence					Effect per 1.000 patients tested		Test accuracy CoE
			Risk of bias	Indirectness	Inconsistency	Imprecision	Publication bias	pre-test probability of 55%	pre-test probability of 85%	
**True positives** (patients with pincer or cam deformities)	1 studies 143 patients	case-control type accuracy study	very serious [a]	serious [b]	not serious	serious [c]	none	61 (39 to 99)	94 (60 to 153)	⨁◯◯◯ VERY LOW
**False negatives** (patients incorrectly classified as not having pincer or cam deformities)								489 (451 to 511)	756 (697 to 790)	
**True negatives** (patients without pincer or cam deformities)	0 studies 0 patients							0 (0 to 0)	0 (0 to 0)	-
**False positives** (patients incorrectly classified as having pincer or cam deformities)								450 (450 to 450)	150 (150 to 150)	

Explanations

a. Inclusion criteria do not exactly match the review question, execution and interpretation of index and reference test not described
b. Test execution and interpretation not described
c. small number of participants

Question: Should IR ROM & FABER be used to diagnose pincer or cam deformities in FAI syndrome?

Sensitivity	0.13 (95% CI: 0.08 to 0.20)
Specificity	-- (95% CI: -- to --)

Prevalences	55%	85%	

Outcome	№ of studies (№ of patients)	Study design	Factors that may decrease certainty of evidence					Effect per 1.000 patients tested		Test accuracy CoE
			Risk of bias	Indirectness	Inconsistency	Imprecision	Publication bias	pre-test probability of 55%	pre-test probability of 85%	
**True positives** (patients with pincer or cam deformities)	1 studies 143 patients	case-control type accuracy study	very serious [a]	serious [b]	not serious	serious [c]	none	72 (44 to 110)	111 (68 to 170)	⨁◯◯◯ VERY LOW
**False negatives** (patients incorrectly classified as not having pincer or cam deformities)								478 (440 to 506)	739 (680 to 782)	
**True negatives** (patients without pincer or cam deformities)	0 studies 0 patients							0 (0 to 0)	0 (0 to 0)	-
**False positives** (patients incorrectly classified as having pincer or cam deformities)								450 (450 to 450)	150 (150 to 150)	

Explanations

a. Inclusion criteria do not exactly match the review question, execution and interpretation of index and reference test not described
b. Test execution and interpretation not described
c. small number of participants

Question: Should IR ROM < 120 be used to diagnose pincer or cam deformities in FAI syndrome?

Sensitivity	0.22 (95% CI: 0.15 to 0.29)
Specificity	-- (95% CI: -- to --)

Prevalences	55%	85%	

Outcome	№ of studies (№ of patients)	Study design	Factors that may decrease certainty of evidence					Effect per 1.000 patients tested		Test accuracy CoE
			Risk of bias	Indirectness	Inconsistency	Imprecision	Publication bias	pre-test probability of 55%	pre-test probability of 85%	
**True positives** (patients with pincer or cam deformities)	1 studies 143 patients	case-control type accuracy study	very serious [a]	serious [b]	not serious	serious [c]	none	121 (83 to 160)	187 (128 to 246)	⨁◯◯◯ VERY LOW
**False negatives** (patients incorrectly classified as not having pincer or cam deformities)								429 (390 to 467)	663 (604 to 722)	
**True negatives** (patients without pincer or cam deformities)	0 studies 0 patients							0 (0 to 0)	0 (0 to 0)	-
**False positives** (patients incorrectly classified as having pincer or cam deformities)								450 (450 to 450)	150 (150 to 150)	

Explanations

a. Inclusion criteria do not exactly match the review question, execution and interpretation of index and reference test not described
b. Test execution and interpretation not described
c. small number of participants

Question: Should IROP be used to diagnose pincer or cam deformities in FAI syndrome?

Sensitivity	1.00 (95% CI: 0.48 to 1.00)
Specificity	0.16 (95% CI: 0.06 to 0.29)

Prevalences	55%	85%	

Outcome	№ of studies (№ of patients)	Study design	Factors that may decrease certainty of evidence					Effect per 1.000 patients tested		Test accuracy CoE
			Risk of bias	Indirectness	Inconsistency	Imprecision	Publication bias	pre-test probability of 55%	pre-test probability of 85%	
**True positives** (patients with pincer or cam deformities)	1 studies 5 patients	cross-sectional (cohort type accuracy study)	very serious [a]	serious [b]	not serious	serious [c]	none	550 (264 to 550)	850 (408 to 850)	⊕◯◯◯ VERY LOW
**False negatives** (patients incorrectly classified as not having pincer or cam deformities)								0 (0 to 286)	0 (0 to 442)	
**True negatives** (patients without pincer or cam deformities)	1 studies 45 patients	cross-sectional (cohort type accuracy study)	very serious [a]	serious [b]	not serious	serious [c]	none	72 (27 to 130)	24 (9 to 44)	⊕◯◯◯ VERY LOW
**False positives** (patients incorrectly classified as having pincer or cam deformities)								378 (320 to 423)	126 (106 to 141)	

Explanations

a. no blinding of assessor, positivity criteria for reference test not declared
b. positivity criteria for reference test not declared
c. very small number of participants

Question: Should log roll test be used to diagnose pincer or cam deformities in FAI syndrome?

Sensitivity	0.30 (95% CI: 0.17 to 0.43)
Specificity	-- (95% CI: -- to --)

Prevalences	55%	85%	

Outcome	№ of studies (№ of patients)	Study design	Factors that may decrease certainty of evidence					Effect per 1.000 patients tested		Test accuracy CoE
			Risk of bias	Indirectness	Inconsistency	Imprecision	Publication bias	pre-test probability of 55%	pre-test probability of 85%	
**True positives** (patients with pincer or cam deformities)	1 studies 40 patients	case-control type accuracy study	very serious [a]	very serious [b]	not serious	serious [c]	none	165 (94 to 237)	255 (145 to 366)	⨁◯◯◯ VERY LOW
**False negatives** (patients incorrectly classified as not having pincer or cam deformities)								385 (313 to 456)	595 (484 to 705)	
**True negatives** (patients without pincer or cam deformities)	0 studies 0 patients							0 (0 to 0)	0 (0 to 0)	-
**False positives** (patients incorrectly classified as having pincer or cam deformities)								450 (450 to 450)	150 (150 to 150)	

Explanations

a. only cases, assessor not blinded, execution of index test not described, exclusion criteria not appropriate
b. execution of index test not described, exclusion criteria not appropriate
c. small number of participants

Question: Should maximal Squat test be used to diagnose cam deformity in FAI syndrome?

Sensitivity	0.75 (95% CI: 0.57 to 0.89)
Specificity	0.41 (95% CI: 0.27 to 0.57)

Prevalences	55%	85%	

Outcome	№ of studies (№ of patients)	Study design	Factors that may decrease certainty of evidence					Effect per 1.000 patients tested		Test accuracy CoE
			Risk of bias	Indirectness	Inconsistency	Imprecision	Publication bias	pre-test probability of 55%	pre-test probability of 85%	
**True positives** (patients with cam deformity)	1 studies 32 patients	cross-sectional (cohort type accuracy study)	not serious	not serious	not serious	serious [a]	none	413 (314 to 490)	638 (484 to 757)	⨁⨁⨁◯ MODERATE
**False negatives** (patients incorrectly classified as not having cam deformity)								137 (60 to 236)	212 (93 to 366)	
**True negatives** (patients without cam deformity)	1 studies 46 patients	cross-sectional (cohort type accuracy study)	not serious	not serious	not serious	serious [a]	none	184 (122 to 256)	62 (41 to 86)	⨁⨁⨁◯ MODERATE
**False positives** (patients incorrectly classified as having cam deformity)								266 (194 to 328)	88 (64 to 109)	

Explanations

a. small number of participants

Question: Should pain predominantly in F/IR be used to diagnose pincer or cam deformities in FAI syndrome?

Sensitivity	0.70 (95% CI: 0.62 to 0.77)
Specificity	0.44 (95% CI: 0.33 to 0.55)

Prevalences	55%	85%	

Outcome	№ of studies (№ of patients)	Study design	Factors that may decrease certainty of evidence					Effect per 1.000 patients tested		Test accuracy CoE
			Risk of bias	Indirectness	Inconsistency	Imprecision	Publication bias	pre-test probability of 55%	pre-test probability of 85%	
**True positives** (patients with pincer or cam deformities)	1 studies 154 patients	cross-sectional (cohort type accuracy study)	serious [a]	not serious	not serious	serious [b]	none	385 (341 to 424)	595 (527 to 655)	⨁⨁◯◯ LOW
**False negatives** (patients incorrectly classified as not having pincer or cam deformities)								165 (126 to 209)	255 (195 to 323)	
**True negatives** (patients without pincer or cam deformities)	1 studies 87 patients	cross-sectional (cohort type accuracy study)	serious [a]	not serious	not serious	serious [b]	none	198 (149 to 248)	66 (50 to 83)	⨁⨁◯◯ LOW
**False positives** (patients incorrectly classified as having pincer or cam deformities)								252 (202 to 301)	84 (67 to 100)	

Explanations

a. see QUADAS 2
b. small number of participants

Question: Should PIT be used to diagnose pincer or cam deformities in FAI syndrome?

Sensitivity	0.11 to 0.36
Specificity	-- to --

Prevalences	55%	85%	

Outcome	№ of studies (№ of patients)	Study design	Factors that may decrease certainty of evidence					Effect per 1.000 patients tested		Test accuracy CoE
			Risk of bias	Indirectness	Inconsistency	Imprecision	Publication bias	pre-test probability of 55%	pre-test probability of 85%	
**True positives** (patients with pincer or cam deformities)	2 studies 190 patients	case-control type accuracy study	very serious [a]	serious [b]	not serious	serious [c]	none	61 to 198	94 to 306	⨁◯◯◯ VERY LOW
**False negatives** (patients incorrectly classified as not having pincer or cam deformities)								352 to 489	544 to 756	
**True negatives** (patients without pincer or cam deformities)	0 studies 0 patients	cross-sectional (cohort type accuracy study)						0 to 0	0 to 0	-
**False positives** (patients incorrectly classified as having pincer or cam deformities)								450 to 450	150 to 150	

Explanations

a. see QUADAS 2, only cases included in one study, examiner was not blinded
b. criteria for index test positivity not stated
c. small number of participants

Question: Should Scour maneuver be used to diagnose pincer or cam deformities in FAI syndrome?

Sensitivity	0.80 (95% CI: 0.28 to 0.99)
Specificity	0.40 (95% CI: 0.26 to 0.56)

Prevalences	55%	85%	

Outcome	№ of studies (№ of patients)	Study design	Factors that may decrease certainty of evidence					Effect per 1.000 patients tested		Test accuracy CoE
			Risk of bias	Indirectness	Inconsistency	Imprecision	Publication bias	pre-test probability of 55%	pre-test probability of 85%	
**True positives** (patients with pincer or cam deformities)	1 studies 5 patients	cross-sectional (cohort type accuracy study)	very serious [a]	serious [b]	not serious	serious [c]	none	440 (154 to 545)	680 (238 to 842)	⨁◯◯◯ VERY LOW
**False negatives** (patients incorrectly classified as not having pincer or cam deformities)								110 (5 to 396)	170 (8 to 612)	
**True negatives** (patients without pincer or cam deformities)	1 studies 45 patients	cross-sectional (cohort type accuracy study)	very serious [a]	serious [b]	not serious	serious [d]	none	180 (117 to 252)	60 (39 to 84)	⨁◯◯◯ VERY LOW
**False positives** (patients incorrectly classified as having pincer or cam deformities)								270 (198 to 333)	90 (66 to 111)	

Explanations

a. no blinding of assessor, positivity criteria for reference test not declared
b. positivity criteria for reference test not declared
c. very small number of participants
d. few patients, CI < 0.5

Question: Should Stinchfield test (RSLR) be used to diagnose pincer or cam deformities in FAI syndrome?

Sensitivity	0.15 to 0.95
Specificity	0.22 to 0.51

Prevalences	55%	85%	

Outcome	№ of studies (№ of patients)	Study design	Factors that may decrease certainty of evidence					Effect per 1.000 patients tested		Test accuracy CoE
			Risk of bias	Indirectness	Inconsistency	Imprecision	Publication bias	pre-test probability of 55%	pre-test probability of 85%	
**True positives** (patients with pincer or cam deformities)	2 studies 46 patients	cohort & case-control type studies	very serious [a]	serious [b]	not serious	serious [c]	none	83 to 523	128 to 808	⨁◯◯◯ VERY LOW
**False negatives** (patients incorrectly classified as not having pincer or cam deformities)								27 to 467	42 to 722	
**True negatives** (patients without pincer or cam deformities)	1 studies 45 patients	cross-sectional (cohort type accuracy study)	serious [d]	serious [e]	not serious	serious [c]	none	99 to 229	33 to 77	⨁◯◯◯ VERY LOW
**False positives** (patients incorrectly classified as having pincer or cam deformities)								221 to 351	73 to 117	

Explanations

a. see QUADAS 2, no blinding of assessor, only cases included in one study
b. positivity of reference test not declared in one study
c. very small number of participants
d. see QUADAS 2, no blinding of assessor, positivity of reference test not declared
e. positivity of reference test not declared
