# Supplementary material for: Diagnostic accuracy of clinical tests for cam or pincer morphology in individuals with suspected FAI syndrome: a systematic review
Source: BMJ Open Sport Exerc Med. 2020 Apr 27;6(1):e000772. doi: 10.1136/bmjsem-2020-000772 (PMC7213881; doi:10.1136/bmjsem-2020-000772)

FABER (Patrick)

Study	TP	FP	FN	TN	Sensitivity (95% CI)	Specificity (95% CI)
Clohisy 2009	52	0	1	0	0.98 [0.90, 1.00]	Not estimable
Maslowski 2010	3	36	2	9	0.60 [0.15, 0.95]	0.20 [0.10, 0.35]
Ratzlaff 2015	53	0	90	0	0.37 [0.29, 0.46]	Not estimable

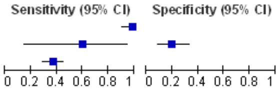

FDT

Study	TP	FP	FN	TN	Sensitivity (95% CI)	Specificity (95% CI)
Philippon 2007	292	0	9	0	0.97 [0.94, 0.99]	Not estimable
Trindade 2018	145	270	26	162	0.85 [0.79, 0.90]	0.38 [0.33, 0.42]

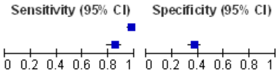

Stinchfield (RSLR)

Study	TP	FP	FN	TN	Sensitivity (95% CI)	Specificity (95% CI)
Clohisy 2009	23	0	18	0	0.56 [0.40, 0.72]	Not estimable
Maslowski 2010	3	29	2	16	0.60 [0.15, 0.95]	0.36 [0.22, 0.51]

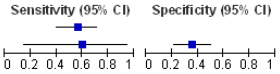

Scour maneuver

Study	TP	FP	FN	TN	Sensitivity (95% CI)	Specificity (95% CI)
Maslowski 2010	4	27	1	18	0.80 [0.28, 0.99]	0.40 [0.26, 0.56]

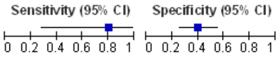

IROP

Study	TP	FP	FN	TN	Sensitivity (95% CI)	Specificity (95% CI)
Maslowski 2010	5	38	0	7	1.00 [0.48, 1.00]	0.16 [0.06, 0.29]

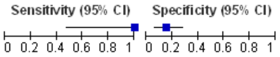

Internal Rotation Pain

Study	TP	FP	FN	TN	Sensitivity (95% CI)	Specificity (95% CI)
Ratzlaff 2015	56	0	87	0	0.39 [0.31, 0.48]	Not estimable

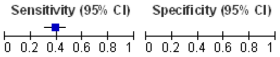

Posterior Impingement

Study	TP	FP	FN	TN	Sensitivity (95% CI)	Specificity (95% CI)
Clohisy 2009	10	0	37	0	0.21 [0.11, 0.36]	Not estimable
Ratzlaff 2015	26	0	117	0	0.18 [0.12, 0.25]	Not estimable

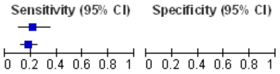

f120 add IR

Study	TP	FP	FN	TN	Sensitivity (95% CI)	Specificity (95% CI)
Ratzlaff 2015	106	0	37	0	0.74 [0.66, 0.81]	Not estimable

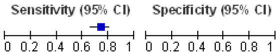

FADIR (AIT, f90 add IR)

Study	TP	FP	FN	TN	Sensitivity (95% CI)	Specificity (95% CI)
Clohisy 2009	47	0	6	0	0.89 [0.77, 0.96]	Not estimable
Philippon 2007	298	0	3	0	0.99 [0.97, 1.00]	Not estimable
Ranawat 2017	107	78	4	10	0.96 [0.91, 0.99]	0.11 [0.06, 0.20]
Ratzlaff 2015	79	0	64	0	0.55 [0.47, 0.64]	Not estimable

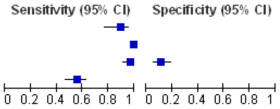

f90 add C

Study	TP	FP	FN	TN	Sensitivity (95% CI)	Specificity (95% CI)
Ratzlaff 2015	47	0	96	0	0.33 [0.25, 0.41]	Not estimable

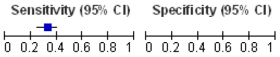

f120 add C

Study	TP	FP	FN	TN	Sensitivity (95% CI)	Specificity (95% CI)
Ratzlaff 2015	86	0	57	0	0.60 [0.52, 0.68]	Not estimable

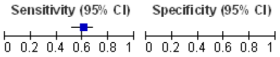

IR ROM < 20

Study	TP	FP	FN	TN	Sensitivity (95% CI)	Specificity (95% CI)
Ratzlaff 2015	31	0	112	0	0.22 [0.15, 0.29]	Not estimable

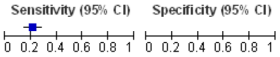

FLEX ROM < 115

Study	TP	FP	FN	TN	Sensitivity (95% CI)	Specificity (95% CI)
Ratzlaff 2015	29	0	114	0	0.20 [0.14, 0.28]	Not estimable

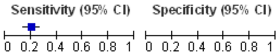

IR ROM & FABER

Study	TP	FP	FN	TN	Sensitivity (95% CI)	Specificity (95% CI)
Ratzlaff 2015	19	0	124	0	0.13 [0.08, 0.20]	Not estimable

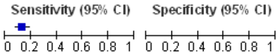

IR ROM & f90 IR

Study	TP	FP	FN	TN	Sensitivity (95% CI)	Specificity (95% CI)
Ratzlaff 2015	16	0	127	0	0.11 [0.07, 0.18]	Not estimable

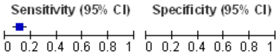

pain predominantly in F/IR

Study	TP	FP	FN	TN	Sensitivity (95% CI)	Specificity (95% CI)
Nogier 2010	108	49	46	38	0.70 [0.62, 0.77]	0.44 [0.33, 0.55]

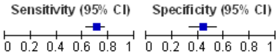

log roll

Study	TP	FP	FN	TN	Sensitivity (95% CI)	Specificity (95% CI)
Clohisy 2009	12	0	28	0	0.30 [0.17, 0.47]	Not estimable

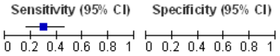

FPAW

Study	TP	FP	FN	TN	Sensitivity (95% CI)	Specificity (95% CI)
Ranawat 2017	68	39	43	49	0.61 [0.52, 0.70]	0.56 [0.45, 0.66]

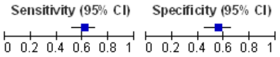

Maximal Squat Test

Study	TP	FP	FN	TN	Sensitivity (95% CI)	Specificity (95% CI)
Ayeni 2014	24	27	8	19	0.75 [0.57, 0.89]	0.41 [0.27, 0.57]

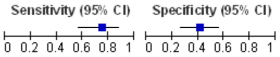

Supplement: Supplementary data [file bmjsem-2020-000772supp004.pdf]
